# Reordering the “World of Things”: The Sociotechnical Imaginary of RFID Tagging and New Geographies of Responsibility

**DOI:** 10.1007/s11948-018-0071-z

**Published:** 2018-10-24

**Authors:** Ulrike Felt, Susanne Öchsner

**Affiliations:** Department of Science and Technology Studies, University of Vienna, Universitätsstraße 7, 1010 Vienna, Austria

**Keywords:** RFID tags, Infrastructure, Responsibility, Video analysis, Sociotechnical imaginaries

## Abstract

The aim of this study is to investigate radio frequency identification (RFID) tagging as a form of sociotechnical experimentation and the kinds of sociotechnical futures at stake in this experimentation. For this purpose, a detailed analysis of a publicly available promotional video by a tag producer for the fashion industry, a sector widely using RFID tags, was analysed in detail. The results of the study indicated that the sociotechnical imaginary of RFID tagging gravitates around the core value of perfect sociotechnical efficiency. This demands a high degree of readiness to engage in standardization efforts, which performs a specific materialized understanding of ethics by other means. Furthermore, the analysis points to the importance of considering the spatiotemporal dimensions in which RFID tags work when reflecting on how this technology matters to society. Finally, the analysis shows a tacit effort to keep RFID technology and thus any questions of responsible innovation confined to the shop floor. However, given the spreading of the use of RFIDs, much wider-ranging considerations are called for.

## Introduction

**Fig. 1:**
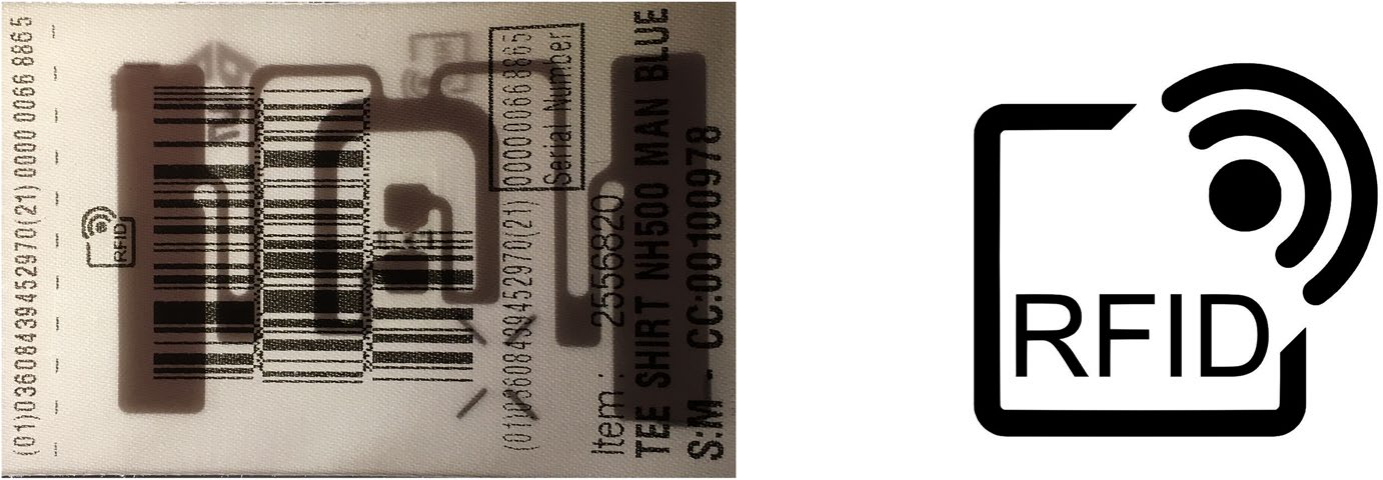
Clothing label with a passive RFID tag shining through (© Ulrike Felt) and icon informing consumers that RFID tags are in use (https://www.aim-d.de/wp-content/uploads/Emblem_rfid-generic.jpg)

When consumers enter major retail stores in the year 2018, they are very likely to unknowingly encounter radio frequency identification (RFID) tags. These tags are invisibly integrated into price tags or placed next to the labels that contain care instructions for clothing (see Fig. 1). Equipped with an electronic scanner that uses radio signals (a reader) and connected to a database, the unique identifier stored in the tag’s chip can disclose considerable information about the product: the route it travelled from its place of production to the store, its exact features, and much more. The RFID tag also enables the tracking of items inside the store—from storage, to the shelves, to different places in the shop.^1^ Consumers who buy tagged items using a credit or loyalty card tacitly enable the potential connection of their name to the items they have purchased. Leaving the store, the tags nested in the purchased items accompany the consumers, and every reader a consumer encounters can access the unique identifier on the chip. As there is no connection to the initial database, the reader cannot access the product information; however, the tag identifier allows the tracking of a specific item’s movement. As more stores begin to use RFID tags, more readers will be available, and more movement profiles of things—and the people “attached” to them—will be possible.

This short story is the entry point to a complex issue that is often also captured by the label “internet of things,” with RFID being a core enabling technology. The above example sets the stage for investigating the potentially conflict-laden coexistence of two expectations in contemporary societies. On the one hand, the dream of a perfectly ordered world is, as science and technology studies (STS) scholar John Law put it, central to modernity: “If our lives, our organizations, […] or our societies were ‘properly ordered’ then all would be well. And we take that such ordering is possible, at least some of the time”. If ordering does not work out, or if a too-high degree of complexity is encountered, he continues, “we tend to treat it as distraction. […] Or we think of it as evidence of failure” (1994, p. 5). The proliferation of information and communication technologies (ICTs) has come to play a key role in the translation of this dream into information infrastructures. On the other hand, citizens living in democratic societies have rather high expectations in regard to the protection of their privacy—particularly related to the growing emphasis on collecting data when ordering the world. It is exactly at this point of encounter between the two expectations—well-functioning order through ICTs and privacy—that the analysis presented in this article is situated.

The introduction of RFID tags is undoubtedly a technological effort to realize this dream of perfect order and it represents an interesting case of a real-world experiment (Gross and Krohn 2005) in which society becomes the quasi laboratory (Krohn and Weyer 1994). This effort means that human subjects are exposed to different kinds of technological and social interventions with unclear or unknown long-term impacts. At the same time, in contemporary innovation-oriented societies, “social practices increasingly present themselves as experiments” and citizens are expected to show “a willingness to remain open to new forms of experience” (Gross and Krohn 2005)—in short: societies are expected to embrace the new. Yet, this also raises a number of questions: Who has the power to design and perform such experiments? Who has a voice when developing the protocols for such experiments? How is informed consent assured in such highly complex and rather invisible sociotechnical infrastructures? And, what are the value systems used to decide the success or failure of such real-world experiments? However, above all, as will be argued, the question of responsibility needs to be posed in new ways (Callon et al. 2009; Felt et al. 2007).

In particular, small and relatively cheap (a few cents per chip) passive RFID tags have captured the imagination of future use(r)s and have, in recent years, already conquered large segments of the retail market (e.g., clothing, shoes, pharmaceuticals), leading to the sale of approximately 10 billion tags in 2017. An important promise is made to retailers if they use RFID technology: they not only can follow their products from production to sale but also “can drive higher sales by making sure they have the right product on the shelf, in the correct size and colour, and at the same time lower their inventory costs due to a more accurate supply chain”.^2^ Indeed, a new sociotechnical infrastructure is imagined and put in place to provide “the undergirding of modern societies” (Larkin 2013, p. 328). It assembles tags, moving items, ICT components, databanks, business plans, and all kinds of actors from producers, retailers, consumers, regulators and many more. In the case at hand, the infrastructure provides the architecture for the circulation of information on goods, creates new ways of realizing and handling the flow of goods, and defines the temporalities in this specific environment. It also brings about new types of vulnerabilities. RFID infrastructures do nothing less than “generate the ambient environment of everyday life” (Larkin 2013, p. 336) for many customers and workers all along the chain of the production and distribution of goods; in other words, they collect, store and make retrievable information concerning features, trajectory and location of goods. The realization of RFID tagging of things seems to be well on its way. Analysts have pointed to the threats that RFID systems pose to privacy and data protection, both for workers in environments that use the technology and for consumers (Fisher and Monahan 2008; Glasser et al. 2007; Lockton and Rosenberg 2005; Wasieleski and Gal-Or 2008), and the debate is still open.

The following analysis takes a step back. Instead of investigating concrete application environments and the new information relations that are created, the aim is to reach a better understanding of the potential futures that are imagined (and thereby produced) through the deployment of a major sociotechnical infrastructure (Larkin 2013) with RFID tagging at its core. This analysis will focus on (1) the visions for a desirable future realized through RFID technologies “sold” to potential clients who should create and fully embrace RFID-tagged environments and (2) the way in which issues of responsibility are/should be addressed in those environments. To accomplish this goal, an in-depth analysis of a promotional video for a company (DETEGO) selling such RFID solutions in the fashion industry—one of the sites where the use of RFID systems is spreading very quickly—will be performed. Understanding these intertwined issues will allow the unpacking of the often tacit assumptions and values that are embedded in and expressed through specific technological realizations. It will further offer insights into the futures that are to be realized through the deployment of RFID technologies. Finally, this unpacking prompts questions that extend far beyond a narrow focus on how to govern specific applications already in place.

## Sociotechnical Projects as Future-Making Agents

When investigating the development and implementation of information infrastructures that are based on RFID technologies, it is essential to move away from focusing on the mere technological capacities of RFID tags, readers or their material realization. Rather, one must look into the wider sociotechnical imaginations embedded in and performed through such large-scale technological infrastructure projects. This viewpoint is essential because, as the social and cultural anthropologist Brian Larkin reminds us, infrastructures always “emerge out of and store within them forms of desire and fantasy” (Larkin 2013, p. 329). In that sense, it is not merely the technological features and possibilities that are of interest to the analyst but also how infrastructures participate—in powerful ways—in efforts to colonize the future (Giddens 1991), conceptualizing it as open “to exploration and exploitation, calculation and control” (Adam and Groves 2007, p. 2). Such visions encoded in technological realizations present projections not only of “what is attainable (…) but also of how life ought, and ought not, to be lived” (Jasanoff and Kim 2015). Making a film to showcase a technology’s potential—as in the film at the core of the following analysis—is one locus for producing such a vision of an infrastructure and the kind of future it is expected to bring into being. Such an effort could be seen as aiming to contribute to the establishment of what STS scholars Sheila Jasanoff and Sang-Hyun Kim (2015) call a “sociotechnical imaginary”. Going beyond a simple vision, which might be more local and promoted by a small group of actors, an imaginary is meant to be much broader and more stable, i.e., a

collectively held, institutionally stabilized, and publicly performed vision of desirable futures, animated by shared understandings of forms of social life and social order attainable through, and supportive of, advances in science and technology. (Jasanoff and Kim 2015, p. 4)

It sensitizes analysts to how profoundly technologies are entangled with technopolitical cultures (Felt et al. 2010), that is, with culturally specific ways of handling technological innovations in society. It also draws attention to the fact that the development of such technological infrastructure projects, on the one hand, and imagined preferred ways of living, value structures, and social order, on the other, are mutually constitutive. Furthermore, it creates awareness that technological projects are never solely about a linear idea of improvement but are profoundly about making choices regarding which societal futures are to be attained and whose values would get realized through certain technologies. The analysis of the video will therefore explore in detail how it contributes to performing and rehearsing (Felt 2015) a desirable future realized through RFID technology.

Looking into large-scale projects of sociotechnical innovation like RFID systems which are meant to shape future developments also calls for engaging with prospective responsibilities. This means reflecting on the purposes and the values related to and performed through RFID systems while at the same time accommodating for uncertainties and ignorance related to how this technology makes its way into this world (Owen et al. 2013). Thus, many aspects of discussing the embedding of RFID technologies into society relate to current European debates on Responsible Research and Innovation (RRI). Emerging technological possibilities are always subject to human choices and, thus, their development can take different directions. Social, economic, legal and not least moral considerations are entangled in these processes of creation, development and implementation. The shape technological development takes is thus never pre-determined but could proceed in one way or another, which in turn has potentially wide-ranging consequences necessitating careful reflection on responsibility issues. In that sense, it is essential to ensure that the ways in which such new information and communication infrastructures take shape carefully reflect human rights and other core values and create societal developments that are morally desirable.

In recent years, a growing body of literature has started to engage with these aspects of governing technological developments in more anticipatory manners, many of which relate to RRI as a conceptual space (Stilgoe and Guston 2017). They all start

from the assumption that contemporary innovation processes cannot be conceived of in an isolated fashion that could be retraced to individual persons, research groups, or even institutions, but that they are embedded in wider societal networks that are comprised of research, engineering, design, marketing, policy-making, and implementation (Leese 2017, p. 1601).

There is a clear idea that issues of responsible choices are never located in a single moment in the design, production or implementation processes but are instead distributed across moments and actor groups.

This thinking connects well to the concept of technological scripts developed by the French STS scholar Madeleine Akrich (1992). She has pointed to the fact that designers of any technology imagine and try to frame an object’s context of use and its user’s behaviour by inscribing their vision of the world into the artefact’s design. Designers imagine users and contexts of use while at the same time delegating responsibility to the artefact, thus creating new geographies of responsibility (Akrich 1992). At the same time, users, i.e., in the case presented here, companies buying RFID technologies, sales personnel working within an RFID infrastructure or consumers navigating shop spaces, might have rather different ideas about the technology and the world inscribed in it, and they might create new role attributions. They struggle with the script, and they might attempt to shift it or even reject it. This situation suggests that the question can never merely be whether a technology such as RFID is “good” or not; rather, one has to look into the many ways in which it is reconfigured and rescripted in practice once being assembled in larger sociotechnical systems.

Therefore, it is essential to consider that such complex technological infrastructures are

highly likely to be comprised of multiple, interacting elements that emerge in multi-year processes, undergo design and marketing choices, eventually become regulated, and might even unfold unprecedented social implications through the ways they become used on an everyday basis. (Leese 2017, p. 1601)

In that sense, looking for intentional transgressions of ethical boundaries by single actors in building technological infrastructures should not be at the core of the analysts’ attention but rather the (potentially) emerging problem zones that are “more likely to result from the unforeseen side effects of *collective action*” (von Schomberg 2013).

## Material and Method of Analysis

In 2015, a software company that develops RFID solutions for the fashion industry released a promotional film to showcase the technology’s potential.^3^ The film was published on the company’s website and via its social media, such as its Twitter, Facebook and YouTube accounts. The video itself is 3 min and 14 s in length; it contains 56 takes in which the viewer is guided through changes (in practices) on the shop floor through the introduction of an RFID system. This video serves as a case study through which to investigate how an important actor in the field imagines and “sells” the potential of RFID applications for the world of fashion and thus the world of tags that is being produced.

As the German communication scholar and videographer Christine Moritz (2011) notes, most current video analyses in the social sciences reduce the element that carries meaning in videos to one of two components and analytically privileges one or the other: image or text. In that sense, analyses either focus on the non-moving image, such as on stills, frames or screens, or on the language within the video, such as words spoken by actors, which can then be transformed into written text and analysed with traditional methods of text interpretation (Moritz 2011). Yet, videos or films are more than a translation of text and singular static images into moving form. The processual choreography of, e.g., image, noise, space, time, movement and light cannot be reduced to a mere addition of text plus image. Videos have to be understood as a particular and distinct system of symbols, signs and expression, and they come equipped with a set of attributes that substantially differ from those of texts. Too great a focus on language alone (and by this the isolation of spoken text within the whole assemblage and its treatment as auditive data) is problematic because it misses out on the multiple entanglements with other elements of the video (such as sound, focus, zoom) and thereby on a fuller conceptualization of meaning (Moritz 2011, pp. 7–14). This argument is supported by the social scientists Jo Reichertz and Carina Englert (2011), who urge a focus on meaningful action as the unit of analysis. In the context of videos, this does not mean the still, i.e., the analysis of one static image after another, but rather the move, which they define as a unit of interaction or communication that has consequences for what is yet to come in the subsequent course of action (Reichertz and Englert 2011, pp. 14–15). Furthermore, they turn the attention to the important distinction between two types of move: the moves of the actors in front of the camera and the moves of the camera as actor (i.e., the moves that a camera makes to see and to show specific things and therefore how those things are shown; Reichertz and Englert 2011, p. 15). In this reading, videos can be understood as an important artefact for reconstructing what is valued and what is not, what is worth showing and what is not.

In addition to the issue of conceptualizing meaning within videos as represented in the move as the unit of analysis, it became clear that in this case there were certain particularities with consequences for the analytic process. First of all, none of the human actors in the video speak beyond saying a number once. Second, apart from a tagging hand, the human actors representing items of clothing are the only humans that appear in the video, so there is also a negligible explicatory level of non-metaphorical humans framing or contextualizing their actions. Third, language appears only in the voiceover and as text overlays. An important task in this paper, therefore, was to make the digital materiality and metaphorical items in the video speak, in the sense of laying out and making sense of the variety of sounds that were used (such as different types of electronic beeps with different meanings and related to different practices, pings, snoring, sonar sounds or the sound of a cash register).

The first step of the analysis was an initial open coding of the video, using the computer program ELAN, and in parallel, structuring the video according to sequential units of meaning to which preliminary headings were attributed to best summarize and capture their meanings. As suggested by Carina Englert and Michael Roslon (2012), these sequential units of meaning were refined more and more in the subsequent course of the analysis. In a second step, a detailed video protocol was produced, which contained the sequence of single takes in order to be able, in a third step, to define the moves (Englert and Roslon 2012) or the “coherent threads of action” that are—following Reichertz and Englert’s approach and in contrast to an analysis of the stills alone—the main units of analysis (Reichertz and Englert 2011). In parallel to this, all relevant elements of action in front of the camera and of the camera itself were described in great detail, which resulted in a score, that is, a protocol of practices or action (Reichertz and Englert 2011, p. 32), which was again coded, with more and more abstract concepts gradually developed from the empirical material. The empirical end product was a condensed and conceptually rich description of ten sequences that illuminate four moments of ordering.

## Empirical Observations

### Four Moments of Reconfiguration

The focus of the following video analysis will be on the work that goes into trying to realize the ideal ordering and the values attached to it. This ordering happens by technologically reconfiguring the shop floor through the introduction of an RFID system. Which logics of ordering—and, accordingly, which values—are being inscribed into this sociotechnical assemblage? What moments of ordering and (re)configuring the infrastructure anew can be identified? What new possibilities do they enable?

This paper identifies four key moments, which show in an exemplary manner what type of ordering work is imagined to be performed by RFID tags. The first moment of ordering is the initial move from untagged disorder and inactivity towards order and control of unruly items through the establishment of an invisible infrastructural ordering (a new geography) of the shop floor. The second moment is devoted to the switching of an item’s ontological status through tagging that enables counting and accounting and produces disciplined activity. The third moment shows the ordering principles upon which the infrastructure is built: the classification of items according to their positions in space and time, as well as the implementation of fine distinctions; all of this is done within a clearly delineated space. Finally, the fourth moment focuses on the liberation of humanized “items” as they leave the seemingly clearly demarcated and ordered shop floor.

### Moment 1: From Untagged Disorder to a New Spatial Order

**Fig. 2:**
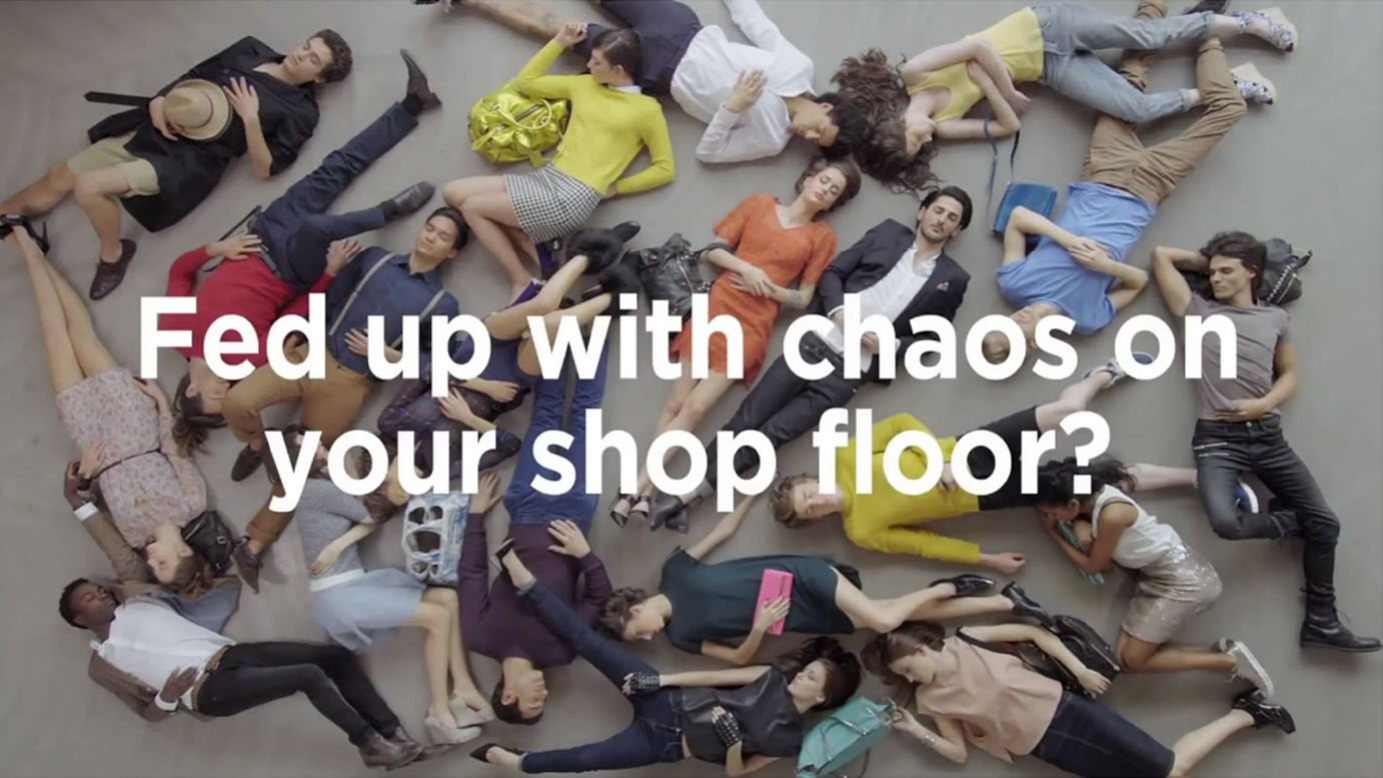
Untagged disorder © DETEGO

The question “*Fed up with chaos on your shop floor?*”, which is shown against the background of a chaotically assembled group of people sleeping on the floor, initiates the spatiotemporal and ontological intervention—through the introduction of an RFID infrastructure—that the viewers of the video are about to witness (Fig. 2). Before this question appeared, all one could see were sleeping people/bodies distributed seemingly randomly on the floor. The careful observer could perceive only two motions: the peacefully rising and falling chests of the sleepers, and some somnambulistic yet erratic movements of their hands. The sleeping state can be read as a visualization of inactivity. The textual level of the male voiceover—“*Managing stock and goods can be hard work*”—connected a number of takes in which the hands of the sleeping bodies were the very focus of the image. This message draws on a specific cultural resource: work that is done with the hands is hard, tiresome and exhausting. Accordingly, work that no longer has to be done with the hands is seen as progress, and this is the first tacit expression of promise of a better future: in a tagged environment, automation sets in and the dire time-and resource-intense work of localizing things, identifying, categorizing and ordering them is handed over to technology. The RFID infrastructure turns into the ‘invisible hand’ that has the capacity to order everything according to predefined variables. This imagination of delegation, however, silences the fact that manual work does not completely disappear in a tagged environment. Employees, for instance, still have to bodily engage with the goods in their store, a task that is indirectly made invisible.

A second element of the sleeping state of the “before” was that observers were positioned right in the middle of the chaos, and it was next to impossible to obtain an overview of what exactly was going on because the camera gave away only extracts, not wholes. If one focused on one item, the rest became invisible. The observer could understand that many more (items, people) were there, but they were blurry, out of focus and unidentifiable. What this alludes to is that in an untagged and therefore unordered world, only little snippets of reality can be in focus—yet with tagging, there would be so much more to “see”, as goes the seductive promise. In the end, the viewer is shown that without technological support, only a very limited section can be overseen and controlled. The complexity that goes beyond what is visible to the naked eye is overwhelming—both physically and emotionally. Trying to bring order into the chaos seems to be a virtually impossible chore. The question thus stages the problem to be solved, but it goes further: it also produces the target group, members of a corporation who have to manage chaos and want to control their shop floor, their supply chain or their inventory processes.

**Fig. 3:**
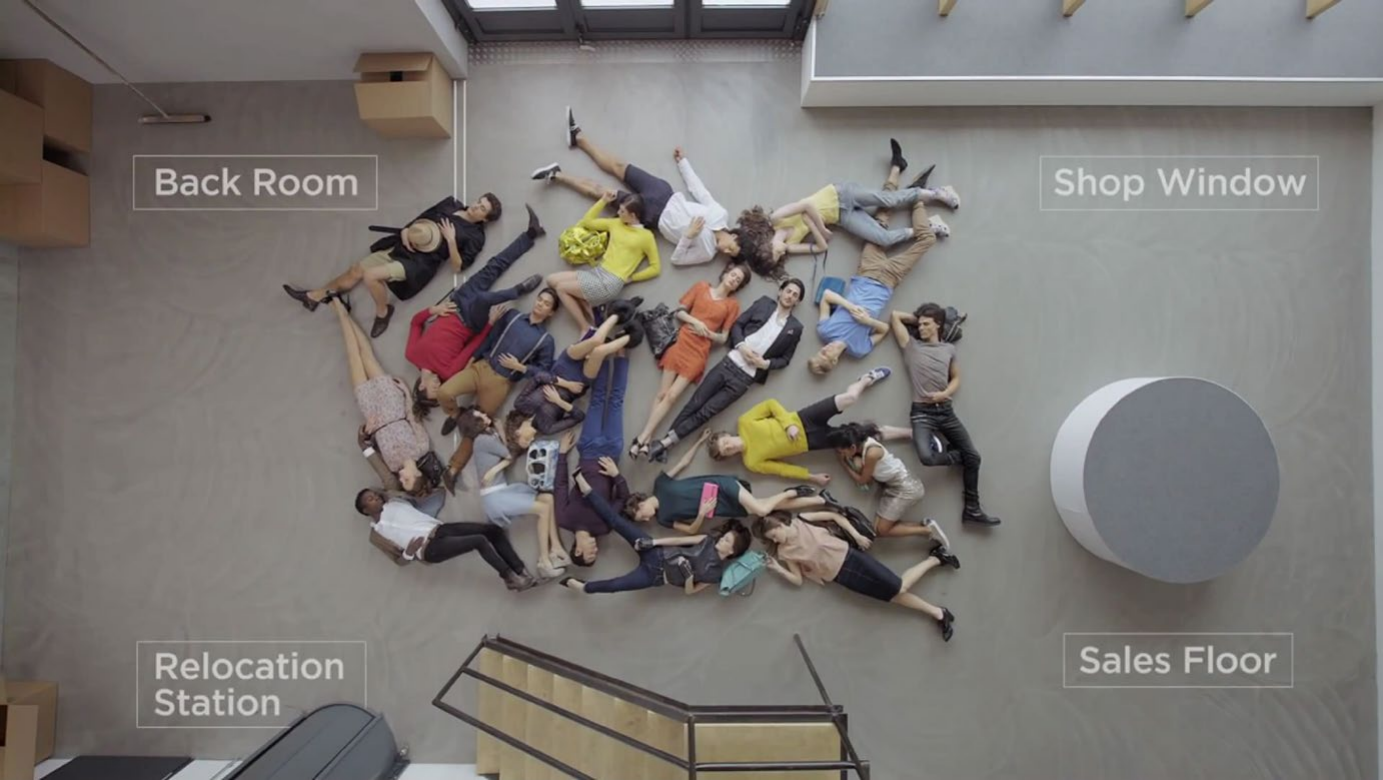
Invisible infrastructural geography © DETEGO

Thus, to transform a disorderly place into a controlled space, a change of perspective is needed. The camera moves towards a view from above. There are some limitations to this viewpoint because if one wants to count and control individual items, they need to lie still and become lifeless and two-dimensional representations. The RFID-enhanced view from above, which is introduced here, displays the view of the infrastructural geography, with four clearly delineated sections of the room: the back room, the shop window, the relocation station and the sales floor (Fig. 3). Additionally, a line is drawn that separates the back room from the sales floor and the shop window, testifying to the constitution of a very specific space through “(structured) orderings of social goods and people in places” (Löw 2008, p. 43). Two practices are important here: the continuous (re)arrangement of different entities (humans and material objects), which the German sociologist Martina Löw (2016) calls spacing, and the practice of synthesis that draws the attention to the connections that can be made between these entities in this sales context.

In the case of RFID infrastructures, this material creation and division of space is invisible to the unknowing eye but is highly relevant to the functioning of the system and the classification of items according to their assigned local positions—i.e., to the creation of the imaginary of a perfectly ordered environment. Furthermore, it produces a within and a beyond, a structured space that is clearly divided in the within and seems demarcated from the beyond. This demarcation of space is consequential for questions of accountability and responsibility, which will be discussed later. And it does something else yet: the establishment of an RFID infrastructure enables the four-dimensional traceability and control of items. If an object is tagged and readers are in place, the object becomes traceable; it is possible to locate it in the space that has been established and to track its movements back and forth through time. At this moment, the people are still untagged humans, motionless and in chaos. For the invisible infrastructural geography to be in full effect, another transformation is needed.

### Moment 2: Switching the Ontological Status

**Fig. 4:**
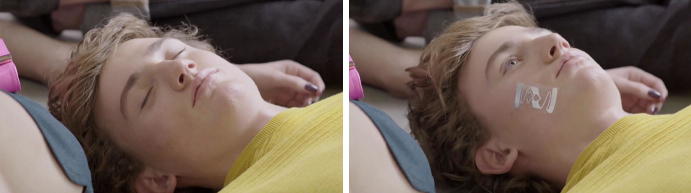
Switching the ontological status through being tagged © DETEGO

The second transformation builds on the just described transformation of the shop floor and establishes an infrastructure that enables the four-dimensional traceability and control of items. Control of items requires communication with them; the bodies thus need to be awake and responsive. At the beginning of Moment 2, the bodies are still not moving and are chaotically distributed on the floor, but something substantial has changed: their eyes are open and their heads and gazes face upward. What has happened? The bodies carrying RFID tags are transformed into readable, and thus arrangeable, items; thus, they changed their ontological status. As soon as there are tags attached to a body, they can potentially be “awakened” (Fig. 4), but they do so only in cooperation with other parts of the infrastructure: the reader with its antenna called “relocation station”. This station is a crucial part of the infrastructure, powering the tags and able to not only localize the tagged body but also collect the information stored on the tag’s chip, i.e., the unique identifier.

Through this process, the lifeless bodies wake up, align their heads and gazes, and become all-attentive. Visually, that awakening process is translated as waves that make a target sign appear—the localizing, awakening and aligning of their gazes are ultimately linked. This transformation confronts the observer with a blurring between humans (active bodies) and sales items on a sales floor—they will thus be referred to as people-items. However, in this space, the differentiation between objects and people does not really matter, as they seem to hold interchangeable properties: traceability, objectification, individual identity.

**Fig. 5:**
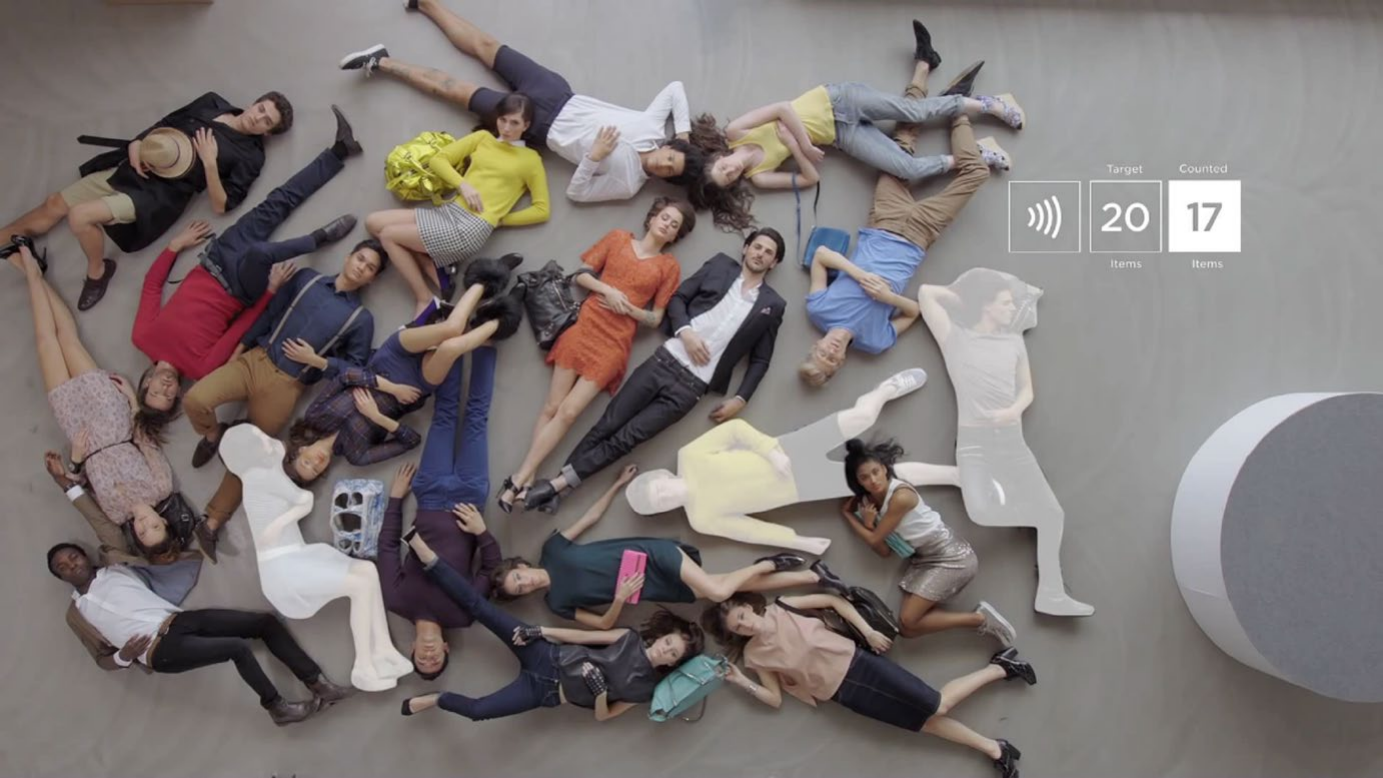
Counting and accounting © DETEGO

If a body is not tagged, i.e., their ontological status did not switch, they remain unreadable and inactive, as is demonstrated in a sequence of the video where three people in the group are represented as transparent (Fig. 5) and motionless until they receive their tag and thus their individual identity. The objectification of human bodies and the tight coupling of tags and infrastructure opens a space in which everything that is present can be counted, managed and controlled. The only things that truly count in such an environment are those that can be read and localized. The others, the ones that cannot be accounted for, are outlaws, grey silhouettes. If something cannot be read and counted, it does not fit in; it is a foreign body that disturbs the imagined and desired purity of the space and needs to be taken care of—either by being removed completely or by being tagged and thus made legible and countable. Having everything tagged is a central value and points to the imagination of perfect order tied to RFID infrastructures.

The switch in ontological status means that the people-items can be linked with the invisible infrastructure and thus be surveilled, tracked and relocated effortlessly. This effortlessness is another central value that is performed in the narrative of the video. In the imagination, the effortless work of relocation and distribution is taken over by the relocation station—the passage point between chaos and order in the seemingly well-demarcated space of the store. Now, the relocation station begins to send out commands to the tagged items, to tell them where to be and where to go, and the people-items begin to move their hands and their feet; they become active and ready to receive and follow commands.

**Fig. 6:**
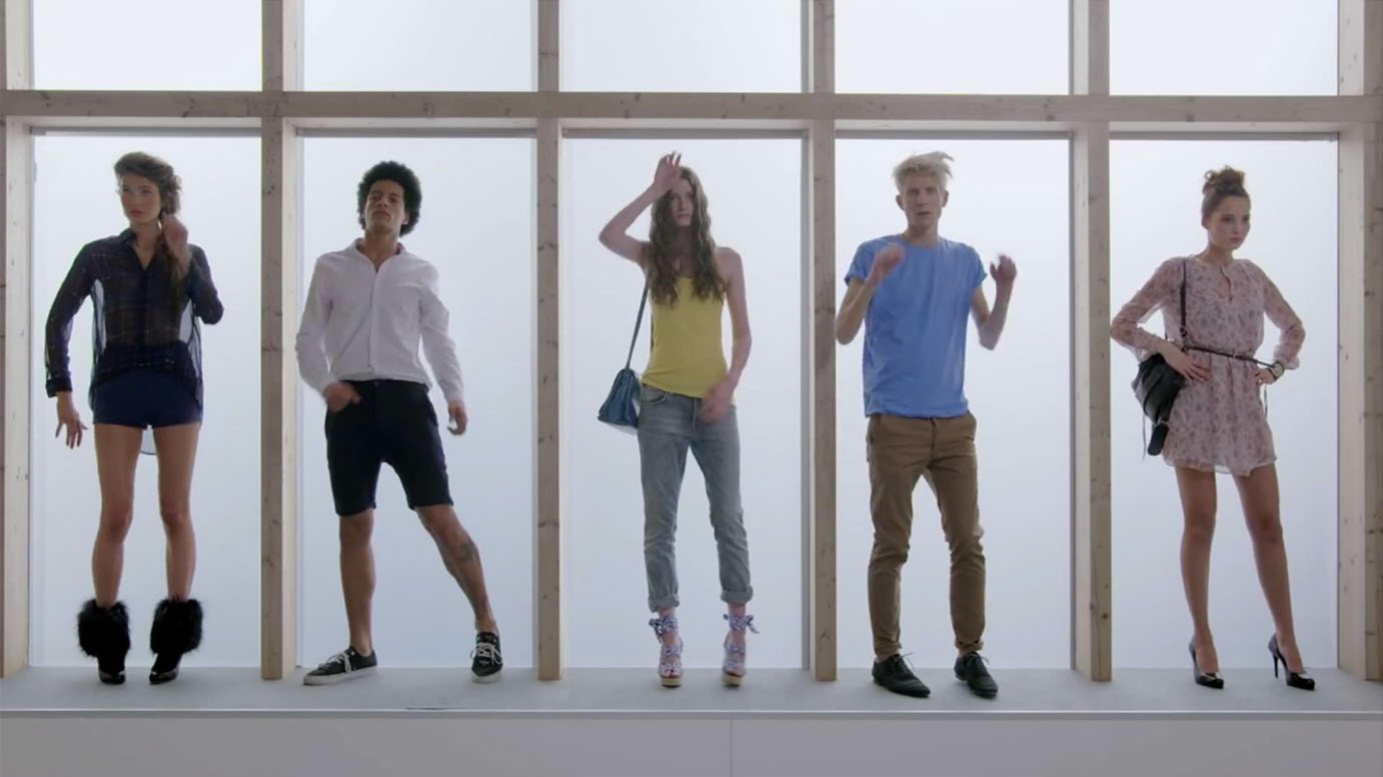
Disciplined activity © DETEGO

However, the shift in ontological status has a second consequence: active people-items begin to move through the room in highly disciplined ways—they act like puppets on invisible strings. What they can and should do in any specific place at a specific point in time seems to be normatively assigned to them. They behave in an orchestrated, compliant way, one that is ordered and strictly follows invisible commands (Fig. 6). Their poses, movements, gestures, facial expressions and even emotions are tamed and linked to the space and time they find themselves in.

The imagination of a perfectly ordered space with docile items, subordinate to the imposed order, is outlined here. The obedience, which is implied in a number of sequences after the relocation station started to transmit activity and location commands, is characterized by the items’ absolute and quick compliance with the invisible order to go to a specific place. This points to an invisible algorithm at work, creating tight alignments of all involved elements, and this includes in the real shop situation human and nonhuman actors. Thus, all actors need to comply with the prescriptions of the system if the whole infrastructure is to function.

In summary, the moment of switching the ontological status has two interrelated transformative consequences. First, a transformation from inactivity to tamed and disciplined activity, and second, a transformation from disorder to order, in which the scope of activity of the items and their appropriate location are defined.

### Moment 3: Ordering and Being Ordered

**Fig. 7:**
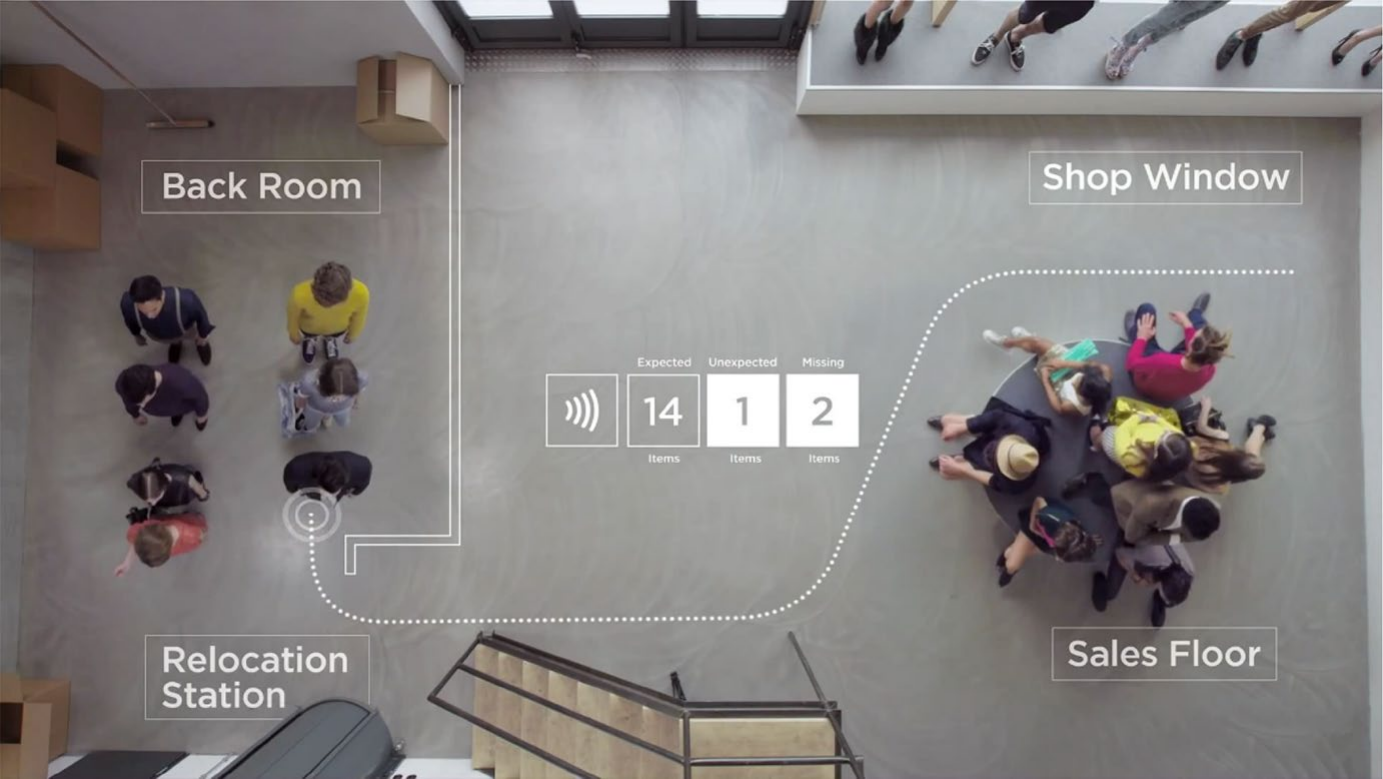
Ordering: being in or out of place © DETEGO

While the items seem to be arranged in a very orderly way and to be well distributed across the topography of the store, three graphic boxes in the middle of the image (Fig. 7) tell a different story.

These boxes form a part of the invisible infrastructure that strongly relies on counting as the foundational practice, as shown in Moment 2. The boxes can show us the count of items within the space, which have been classified into three categories based on their being in or out of place and time. First, there is the standard category of expected items, against which the distribution of objects is tested by means of counting. These items are in the places where they are supposed to be at a given point in time. Then, the first deviation from the standard can be identified, i.e., the category of unexpected items. This category points to a surprise, to something additional that is present within the shop floor, while according to the predefined order, it should not be there. Additionally, there is a second deviation from the standard, a second cause of surprise: the category of missing items. Two items that should be within the space are not. Tagged items are normatively expected to be distributed in an orderly way, which is exactly the purpose of the standardization. They have a particular place in the space–time continuum; this is where they will be found. The category “missing”, thus, is not simply a value-free category—it points to a void in the space where there should be none. “Unexpected” is a value-laden category as well because it points to the divergence from the prescribed order of items within space and time. There simply should be no surprises in a perfectly ordered world. Everything should be known in advance; one has to be able to count on (and with) it. If the space has become impure, it has to be ordered again. From the right side of the image, a pointed line starts to move to the left, accompanied by a sonar sound, searching for the target—and finding it effortlessly. The unexpected and missing items then smoothly move to their correct place, and the prescribed order has thus been reinstated.

This invisible infrastructure has the capacity to clearly categorize any item according to its location at a specific moment in time. The reader-tag-database interaction can even account for very subtle differences between items and thus make extremely fine distinctions, much finer than the naked human eye could ever do. This process is visualized by identical twins (as a representation of similar-or identical-looking items) that can easily be kept apart and then individually endowed with their appropriate place. They comply and walk off in separate directions because they have been given an essential piece of information: their right place. This vision further builds up the imaginary of the magical effortlessness with which the classification and location-assignment can be realized. The imagination stages the RFID infrastructure as enabling deep classification based on fine distinctions and thereby leading to stable forms of purity. This story is, of course, highly seductive, promising perfect order that one can rely on, without ambiguities, without relying on human vision and without the dangers of hidden impurity that one might not be aware of.

While until now, the space and time to be ordered have been meticulously defined and the control of space and time has been represented as all-encompassing, towards the end of the video a boundary is shown to the viewer. A particular transformative place is introduced to point to the spatial boundary of the store: the point of sale that all tagged items shall pass at some point in time and that is signified by a counter and delineated with a red carpet. The point of sale as passage point links the inside to the outside, and it transforms the items from being something that has to be tamed and controlled into something else (see Moment 4). Four items approach the point of sale, walking in a line. Their attributes have changed. Now, the graphic box next to their heads does not show their identifying number any longer but rather an on/off sign and the words “EAS on” (EAS stands for electronic article surveillance). This automatic electronic surveillance becomes relevant only if there is a possibility of the items leaving the store, and it is switched off once they have been read by a sensor at the point of sale. However, the only thing that is being disabled here is the items’ responsiveness to the alarm system; the link to the infrastructure is not severed, as the RFID tags remain on the items. In this imagination, the severing of the link is not necessary because for everything that goes beyond the point of sale, the classifications and ordering principles no longer apply.

### Moment 4: Liberating the Humanized Items

**Fig. 8:**
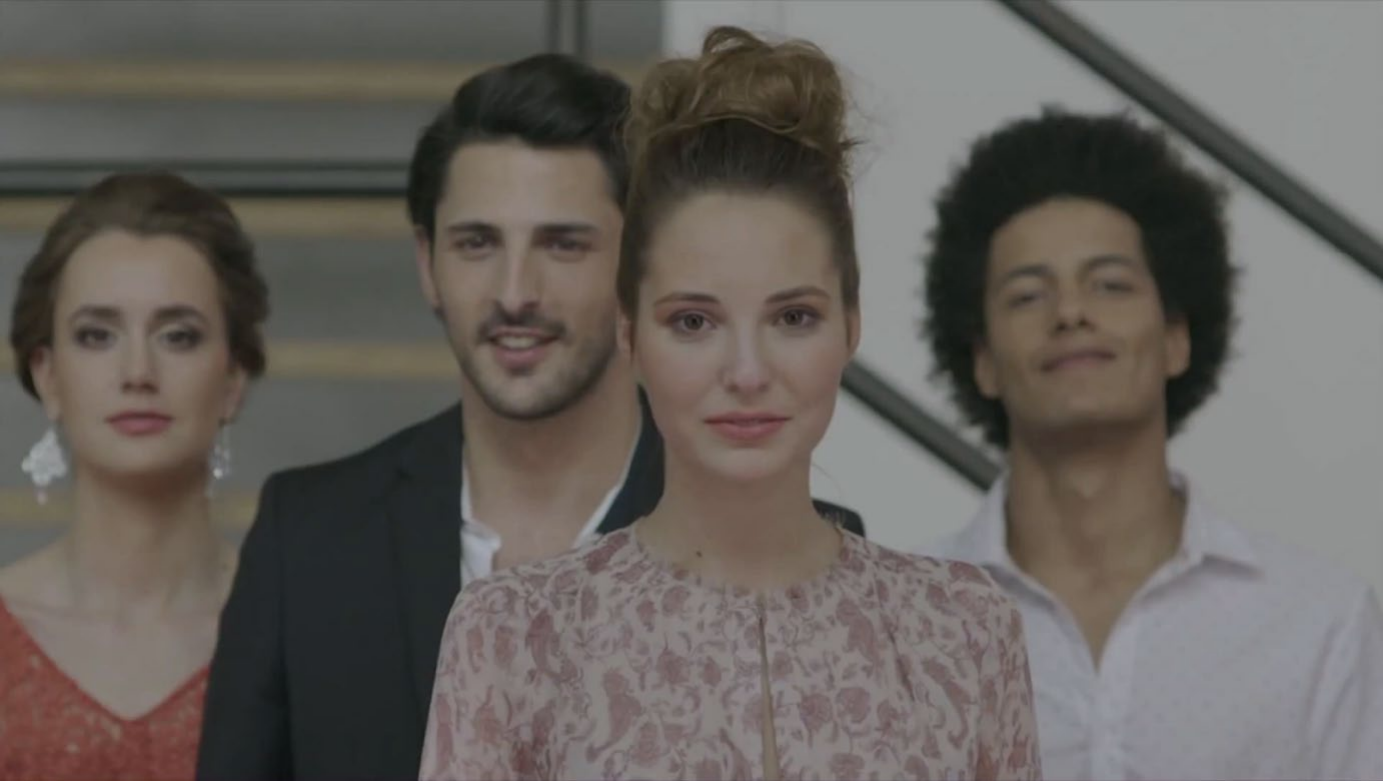
Liberated people-items © DETEGO

**Fig. 9:**
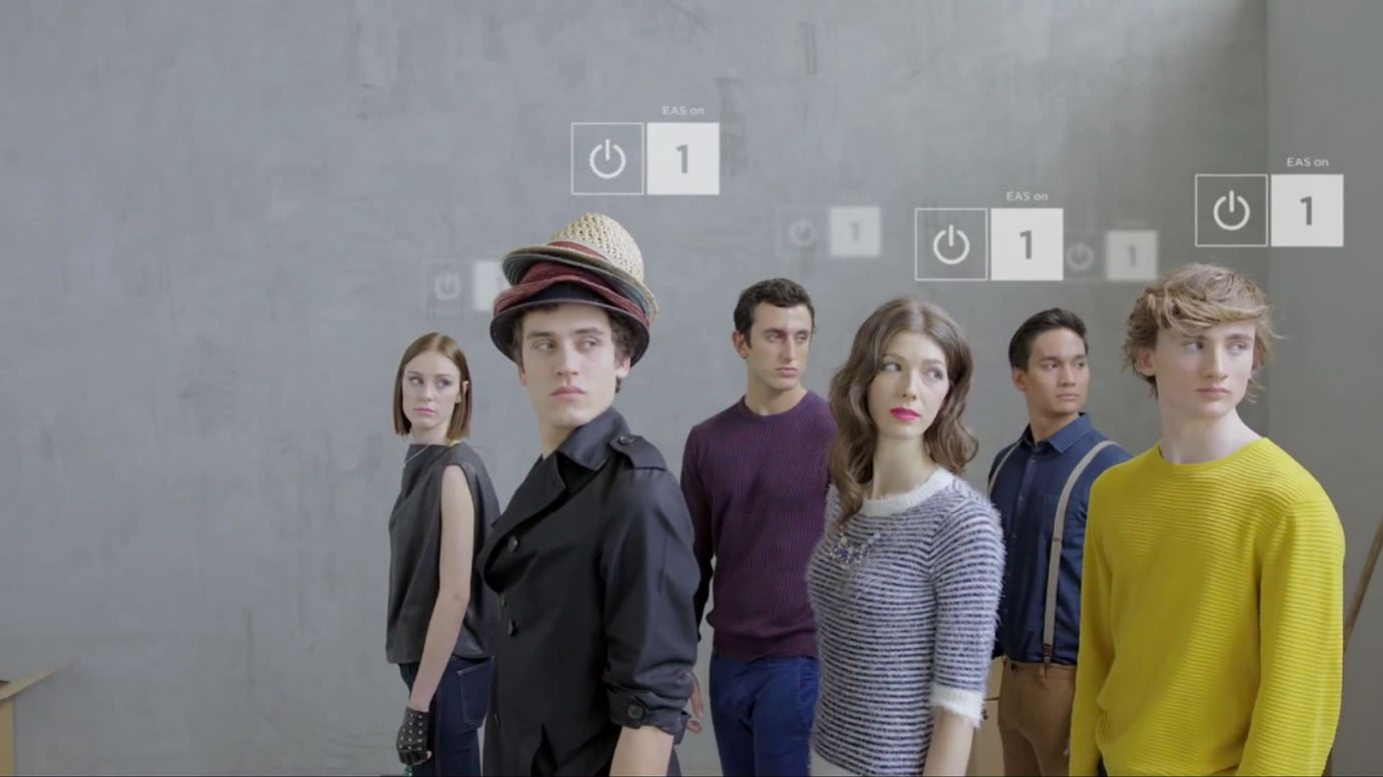
People-items remaining in the store © DETEGO

At the end of the video, a group of four people leaves the delineated space of the store. The comparison of a still of this group with a group staying within its bounds clearly shows that the transgression of the store’s boundary has had consequences (Figs. 8, 9). The items forming the group that stays on the inside change neither their ontological status nor their behaviour. All of the tagged ordered items within the shop remain in place, they do not leave their allocated position or place in line. They are still ordered subject-objects, aligned, linked to a point in space and time, part of a group, tense and sincere silhouettes. While they remain positioned and orderly, they all turn their heads and follow the items leaving the store longingly with their eyes, as if there were a collective understanding that something/someone has left the group: they display some kind of sensing and awareness of other networked items. The image is piercingly bright; all of the items remain in focus and can still be perfectly seen, counted and given instructions.

The items leaving the store, on the other side, have been visually re-transformed into humans—they have been “enabled to leave the store” to use the formulation of the background narrator. They step through the shade into the sun, which for a moment renders their faces blurry. In walking towards the camera, the four of them are no longer equally in focus—one woman is, the other three people move into the background. However, it is also not necessary to have them all in focus; now that they have passed the point of sale, they do not need to be counted and controlled any more. Having left the shop, their classification with regard to space and time does not apply anymore because they are no longer individualized parts of a tamed collective under a regime of disciplined activity and strict compliance with orders. They no longer form a homogenous group with regard to their facial expressions, posture and movements; their gestures and mimics are no longer as concerted and strictly tamed, which—in comparison with the disciplined activity of the items from before—underlines their human individuality. Now that they have passed the threshold of the point of sale, it seems as if they can simply step out of the clearly demarcated space, and in doing so, they look free and relaxed (Fig. 8). The remaining people-items seem to watch those leaving the store with a mixture of envy and sadness (Fig. 9).

What is being completely left out of the narrative here is that the tags remain on the items that have left the store. One sees the tacit construction of the imagination that outside of the shop, the status of being tagged has no consequences because there is no infrastructure in place to which tags could be linked. The information infrastructure ordering the shop space seems to have lost its power; items no longer have to be counted and accounted for. The tag leaving the shop is thus being staged as completely unproblematic. The shop appears as a closed world, well demarcated, in which tags and readers impose perfect order and realize a world that is imagined as fitting the consumer’s expectations of the standard world.

“Let us work our magic. Give your customers extra time and service to accelerate your competitive advantage” is the last message of the narrator before the people-items go back to sleep and the company’s name is shown.

This brings the analyst back to how this story began. What would happen if one considered the messiness of the world outside the shop floor, the multiple overlapping orders created by other systems, the merging of different information systems, and much more?

## Discussion and Conclusions

### Sociotechnical Futures

Studying this promotional video allowed a glimpse into the efforts to construct a sociotechnical imaginary (Jasanoff and Kim 2015) of the infrastructures built around RFID tagging in the case of the fashion industry. It was possible to see how essential it is to not only focus on the RFID tags—the core technology—and their capacity, but to be attentive to the more complex infrastructural project and the publicly performed vision of a desirable future which is embedded into and which comes to life through it. More concretely, the sociotechnical imaginary of this specific RFID based infrastructure has several dimensions specific to it. First is the highly value-laden narrative of the perfect efficiency of processes in the handling of goods. This is closely intertwined with the ideal of a better future that can be brought about through management, full accountability, control and order. And it comes simultaneously with a wider societal benefit narrative: always knowing what is available, and when and where; a perfect match between producers and consumers; effortless order and continuous control—a benefit for all if RFID tags were widely implemented.

The imaginary, secondly, builds on the idea of the unique, clearly standardized and classifiable good that can be traced throughout its entire life and always has a clear space–time position where it belongs. This supports the ideal not only of perfect surveillance and order but also of transparency. This part of the imaginary is closely tied to the belief in society’s ability to carefully shape and control societal futures through technological innovation. Rolling out a system of manufacturing, transport and distribution that can be controlled in real-time through RFID tagging thus supports the idea that in today’s world an improved capacity to anticipate and work with near-future needs is required and possible. User and producer needs are thus seen as entangled through this technological possibility in a quasi-perfect manner.

Thirdly, the choreography of moves of people-items on the shop floor as well as the distinction of items as being in or out of place (Douglas 2002/1966) is no longer a task to be achieved by human actors, but is, through the introduction of RFID tagging, increasingly delegated to algorithms, which in turn promise flawless work. This means that the enactment of perfect order and control is (and needs to be) delegated to and embedded into the technological infrastructure itself. In this part, the imaginary human workers are visually and discursively absent while simultaneously pointing to their imperfection and the kind of adaptive work that would need to be done by humans to fit into this perfect world performed through the internet of things.

It thus seems clear that the deployment of RFID tagging does not solely support a technological process of production and distribution. The video clearly captures and publicly performs a vision of a desirable future that builds on an assumed shared understanding of social life and social order supported by RFID innovations—one of perfect sociotechnical efficiency.

### The Invisible Hand: A World of Standard Operations

To be able to realize this imagination of future sociotechnical worlds, the video points to the fact that RFID tagging is closely tied to the development of invisible algorithms which “care” to establish and keep the sociotechnical imaginary of RFID-tagged environments in place. Algorithms thus become an essential part in the future-making activities of this sociotechnical infrastructure. In particular, in Moments 2 and 3, the viewer encounters the algorithm starting to order the space, yet it always remains black-boxed, unquestionable. It remains tacit how and why items are moved to particular locations and how the decisions of whether something is in or out of place are made. The viewer only witnesses the transformative effects of how the space and people-items are reorganized and controlled. Additionally, there is a tacit promise: more operations per time than humans could perform, reduced costs through controlling the whole chain of production, distribution and consumption and potentially, an ideal shopping environment for customers. Simultaneously, the observations offered here relate directly to the diagnosis of multiple social scientists, stressing that algorithms “play an ever-increasing role in the exercise of power, a means through which to automate the disciplining and controlling of societies and to increase the efficiency of capital accumulation” (Kitchin 2017, p. 15). As outlined earlier, it is simultaneously essential to not think of algorithms as only a purely technical translation of simple human actions into a mode of automated operation. Algorithms are inherently shaped not only by a plethora of decisions, be they political, economic, social or ideological, but also by the materialities of hardware (e.g., what RFID tags or readers can do) and the infrastructure that is expected to enact their instructions. This clearly connects to the efforts of standardization, which are part and parcel of any automated environment.

Introducing standards aims to render the world equivalent across cultures, time, and geography, which means that the introduction of RFID tags is never solely a local endeavour but is meant to precisely create and sustain these new networks of information. With standards being “the recipes for making reality” (Busch 2011), they are the essential basis for implementing, prescribing and stabilizing certain (value) orders. The world and its constituents made visible in the video seem to be neatly standardized and categorized. They can be seen as in or out of place, they can be (re)grouped, or they can undertake many more ordering exercises. Reflecting on the standards and classification work that come with the deployment of RFID tags means being attentive to the fact that “each standard and each category valorizes some point of view and silences another” (Bowker and Star 1999, p. 5). They transport a specific vision of what is technologically possible and good for all—without ever speaking about the exact addressees. Standards and categories are expressions of power, i.e., they serve to empower some and potentially disempower others. However, the STS scholars Geoffrey Bowker and Leigh Star make us also aware that, in today’s world, standardization and classification have become inescapable practices and that this is by no means an inherently problematic thing. However, they point to the fact that standards and classifications are always tied to “an ethical choice” (Bowker and Star 1999, p. 5).

To summarize, what the performance of RFID tagging in the video has shown is a techno-moral project of standardization of both people and things (Busch 2011). Standardization and classification as well as control through algorithmic power that drives the choreography of the movement of people-items is exercised only within the confines of the shop floor, and thus any problem in terms of surveillance or privacy issues is also constructed as limited to this context. The algorithm itself remains invisible, unaddressed and thus widely untouchable. What the video thus shows, is that standards as well as the algorithms that use and impose them should never be considered simply as a technical project but as one that is at the same time social, moral, legal, and ontological—and all this in one go. Realizing technological systems based on RFID technology thus can be seen as an effort to materialize morality (Verbeek 2006), i.e., of doing ethics by other means. This, evidently, opens up questions of responsibility in new ways—the object to be scrutinized remains invisible.

### Experimentation, Laboratory and the Limits of Responsibility

To fully grasp the experimental character of the design and implementation of RFID tagging infrastructures and to be able to address the question of responsibility, it is important not only to highlight the open-endedness and tentativeness of the interventions as pointed to in the beginning of this paper. Rather, it is necessary to simultaneously ask where the “laboratory walls” are imagined to be, i.e., to ask what the space looks like in which the initial experiments would be taking place and thus who becomes a part and in which ways in this experiment. The understanding of the “laboratory” in the context of this analysis was inspired by the STS scholar Michael Guggenheim’s (2012) argument that a laboratory must not necessarily be a well designated closed space but can also be seen as “a procedure that […] results in a space with the properties to separate controlled inside from uncontrolled outside.” (2012, p. 101) This definition allows a reflection on how the space for the RFID experiment is made, how it is imagined and practiced, and how this ability to define experimental spaces becomes an expression of power (Lefèbvre 1991; Löw 2016). Confining experiments to lab spaces imagined as closed and clearly separated from society at large has long allowed putting in place a specific regime of responsibilities. Researchers are allowed to engage in experiments inside the lab as long as nothing escapes into society at large. In the case at hand, it will be essential to ask whether there are such clear-cut laboratory walls—i.e., can the shop floor be conceptualized as a laboratory?—and whether the humans affected identify themselves as experimental subjects (both clients and sales personnel) and what all this means in terms of responsibility for different actors in RFID systems.

This analysis of the video shows the (re)making and (re)thinking of a specific space: the shop floor. Investigating material spatial practices (Lefèbvre 1991) performed through the use of RFIDs means to draw the viewers’ attention to the physical and material flows, transfers, and interactions. Looking out for the signs, codes, and circulating information then allows for new ways of grasping and make sense of the shop floor. Finally, it became clear how this technology not only brings about novel forms of representing space but also allows for reimagining and re-performing it in ways unthinkable before. Making space and ordering space never only means reassembling human and nonhuman entities; it also redistributes agency and recreates networks and new meanings that can be stabilized.

This spatial ordering work therefore has to be seen as a deeply political activity, displaying and reconfiguring power relations. The control of space, to be achieved through the implementation of RFID systems can, then, from this perspective, following Henri Lefèbvre (1991) and Martina Löw (2008), be considered as a specific expression of the capitalist mode of production. Entering this new space and leaving it becomes an important moment in this technological narrative. While being controlled within the space of the shop floor, once they leave, people-items are staged as liberated (see Moment 4). They seem to have escaped from the reading devices and the ordering force they transmit with the help of databases operating invisibly on the backstage.

### Narrowing Responsibility to the Shop Floor?

Finally, both the performance of the specific sociotechnical imaginary as well as the spatial performance together allow a specific “geography of responsibilities” (Akrich 1992, p. 207), i.e., a specific distribution of responsibility, to unfold. It is a geography that seems well contained to the shop floor, the video suggesting that RFID infrastructures are active only in this well-confined space. This suggestion performs work on the level of potential privacy concerns that might be raised in the context of RFID tagging. Through showing a form of technological containment to the shop floor, ethical concerns also become limited in space and time. In the video, this is captured by the image of liberation when people-items are turned into people again and can leave the time–space of the shop and freely move in the “outside world” (Moment 4). This narrative of a spatiotemporal limitation allows RFID tagging to unfold a specific idea of responsibility, one where the balance between benefits and concerns is to be assessed only in the rather narrow space of the shop floor. In that sense, the shop floor is turned into a laboratory in which controlled experiments—with a better ordering of the world—are made, pretendedly without consequences for the world outside. Therefore, it appears sufficient to care about the rights of those working on the shop floor. Clients are often rather vaguely informed about the fact that RFID tags are used in a shop, as here the responsibility is seen only as short-term—until those clients leave the shop with the RFID-tagged goods they bought. RFID tagging is thus an excellent example of a wider challenge to responsible innovation. Does it make sense to discuss responsibility issues in relation to RFID tagging in terms of the limited space–time frame (the shop-floor or any other space) or does it need a much broader understanding of what is at stake once this technology is widely used? This paper definitely presents an argument for the latter.

## Footnotes

1 This paper addresses the use of so-called passive RFID tags, which consist of a mini chip and an antenna. They do not possess their own power source (as compared to active tags which constantly transfer information) and thus need to be activated by an external reader. RFID tags are deployed in many contexts (e.g., access control to buildings, health care instruments to track use frequency and their location, book tracking in libraries, supply chain management tracing objects), encountering different user groups. They have increasingly become an important source of information in contemporary societies.

2 https://www.rfidjournal.com/purchaseaccess?type=Article&id=15491&r=%2Farticles%2Fview%3F15491.

3 The video produced by the company DETEGO can be found at https://www.youtube.com/watch?v=ejB_F8FEoWU&t=53s.
